# Supplementary material for: A comprehensive non-redundant gene catalog reveals extensive within-community intraspecies diversity in the human vagina
Source: Nat Commun. 2020 Feb 26;11:940. doi: 10.1038/s41467-020-14677-3 (PMC7044274; doi:10.1038/s41467-020-14677-3)

**Supplementary material on gene richness definition of vaginal microbiome**

For gene count category and analysis, the included 264 vaginal metagenomes were classified as either having a high gene count (>10,000 non-redundant genes) or low gene count (<10,000 non-redundant genes). The VIRGO non-redundant genes were then annotated as either being a high gene count gene or low gene count gene if the gene was preferentially identified (at least 95%) in high or low gene count metagenomes. The log ratio of genes of a species being in either high or low gene count metagenomes across the 264 vaginal metagenomes was calculated for all species with at least 0.1% abundance and at least 100 genes in either HGC or LGC groups. The species with more than 4 times more abundant (in logarithm 2 scale) in a category (either HGC or LGC) were considered showing preference in one of the categories.

We observed gene richness is characteristic of vaginal microbiomes. Gene richness, calculated as number of non-redundant genes, has been adapted as the proxy of genetic diversity based on community gene content, and more recently, as community-level biomarker in gut quantitative metagenomics studies ^45,46^. We applied this paradigm to vaginal metagenomes included in VIRGO and defined high gene count (HGC) vaginal communities as those that contained >10,000 non-redundant genes and low gene count (LGC) vaginal communities as those that contained ≤ 10,000 non-redundant genes. As expected, HGC communities had a significantly higher number of non-redundant genes (29,898±1,025) than LGC communities (4,920±151.6), however these types of communities also showed differences in their functional makeup. The LGC communities were found to be enriched for genes related to carbohydrate transport and metabolism, as well as those involved in transcription, while HGC communities were found to be enriched in genes related to intracellular trafficking, secretion, and vesicular transport, including coenzyme transport and metabolism (**Supplementary Fig. 10**). We also found that *Lactobacillus*-dominated communities were typically categorized as LGC (82.9%) and *Lactobacillus*-deficient communities as HGC (88.3%) (**Fig. 4a**). However, this was not always the case, most notably, *L. iners*-dominated communities were classified as HGC 21.7% of the time, the highest percentage among all *Lactobacillus*-dominated communities. In fact, *L. iners-*dominated communities (7,803±6,973) generally had a greater gene richness than *L. crispatus*-dominated (5,409±3,392), *L. gasseri*-dominated (3,909±2,761), and *L. jensenii*-dominated (3,990±3,230) communities. Further, *L. iners* in HGC communities and *L. iners* in LGC communities show distinct functional makeup (**Supplementary Fig. 11**). Similarly, not all *Lactobacillus*-deficient communities were classified as HGC—11.7% of these communities were identified as LGC. This includes communities with a high abundance of *G. vaginalis,* whose gene richness varied between 7,689±1,700 in LGC and 16,887±566 in HGC communities.

In addition to being a characteristic of individual communities, individual genes based on their observed preference for either HGC or LGC communities. Using data from the 264 vaginal metagenomes, we classified each non-redundant gene as either an HGC or LGC gene if ≥95% its occurrences were in HGC or LGC communities, respectively. Genes that did not meet this criterion were annotated as having no preference. These gene richness annotations were included for each non-redundant gene in VIRGO. For example, 84.1%, 53.3%, 60.5% of top prevalent tryptophan biosynthesis genes in VIRGO, tryptophanase (TNAA), tryptophan synthase beta chain (TRPB), and tryptophanyl-tRNA synthetase (TRPS), are HGC genes, while 0%, 0%, and 7.0% are LGC genes (**Additional file1: Table 9)**. Given the top most affiliated taxonomic groups for these tryptophan biosynthesis genes were identified as *G. vaginalis*, *A. vaginae*, *M. mulieris* (**Supplementary Fig. 12)** our result indicates tryptophan biosynthesis genes are most prevalent in BV-associated bacteria of high gene richness vaginal communities, agreeing with recent studies ^47,48^.

Using these gene annotations, we were further able to evaluate whether a vaginal bacterial species’ genes were overrepresented as being HGC or LGC (**Fig. 4b**). *Lactobacillus* spp., particularly *L. crispatus*, *L. jensenii, L. gasseri, L. vaginalis,* were observed to be highly overrepresented in LGC communities. On the other hand, genes belonging to many other BV-associated species, specifically *P. timonensis*, *P. buccalis, P. amnii, M. mulieris*, *Mageeibacillus indolicus*, *Porphyromonas uenonis, P. harei, Anaerococcus tetradius,* *M. curtisii*, were overrepresented in HGC. These results demonstrate how gene richness-based annotations provide and added dimension to our understanding of the genetic basis of the biological processes that drive vaginal microbiomes.

Overall, using VIRGO, we observed that vaginal metagenomes varied in gene richness, with some communities having more non-redundant genes than others. Gene richness has been found to be indicative of the pathophysiological state of the gut microbiome in studies of obesity ^53^, dietary intervention ^54^, type II diabetes ^55^, and inflammation and metabolic disease ^56^. We adapted the concept of gene richness as a characterization of community gene content and defined an analogous definition for the vaginal microbiome. An outstanding difference in gene richness was observed between *Lactobacillus*-dominated and *Lactobacillus*-deficient communities. Approximately 85% of communities with a high relative abundance of *Lactobacillus* sp., had a low gene richness across the community, whereas *Lactobacillus*-deficient communities were more likely to have a high gene richness. However, around 22% of *Lactobacillus*-dominated communities did have high gene richness and 12% of *Lactobacillus*-deficient communities had low gene richness. It may be that gene richness category, when combined with community state types, provides a useful and, ecologically relevant, categorization of vaginal community states. For example, it is envisioned that a subject that has a *Lactobacillus*-dominated community with high gene richness is at a higher risk of switching to a dysbiotic state than one whose community is dominated by *Lactobacillus* but with low gene richness. In such case, VIRGO provides the analytical suite needed to test this and other hypotheses relating gene richness to the ecology of the vaginal microbiome.

**Supplementary Figures:**
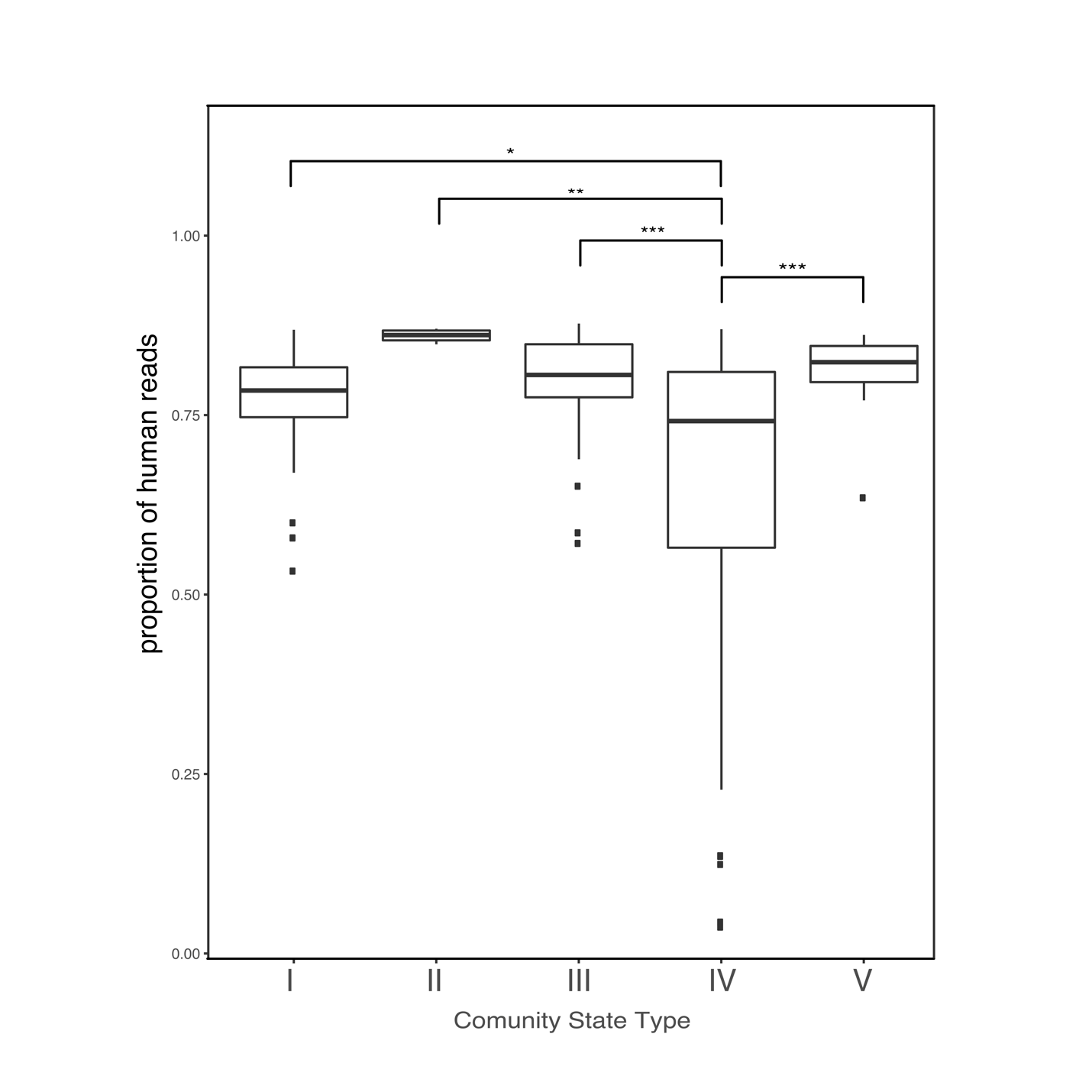
**Supplementary Fig. 1** | Boxplot of the proportion of sequencing reads after removing human contaminates from the samples between different Community State Types (CSTs). CSTs were defined as previously according to the composition and structure of the microbial community [29]. Plotted are interquartile ranges (IQRs, boxes), medians (line in box), and mean (red diamond). Significance value was calculated using Wilcoxon rank sum test using *ggsignif* R package [99]. Star sign (*) denotes the level of significance.


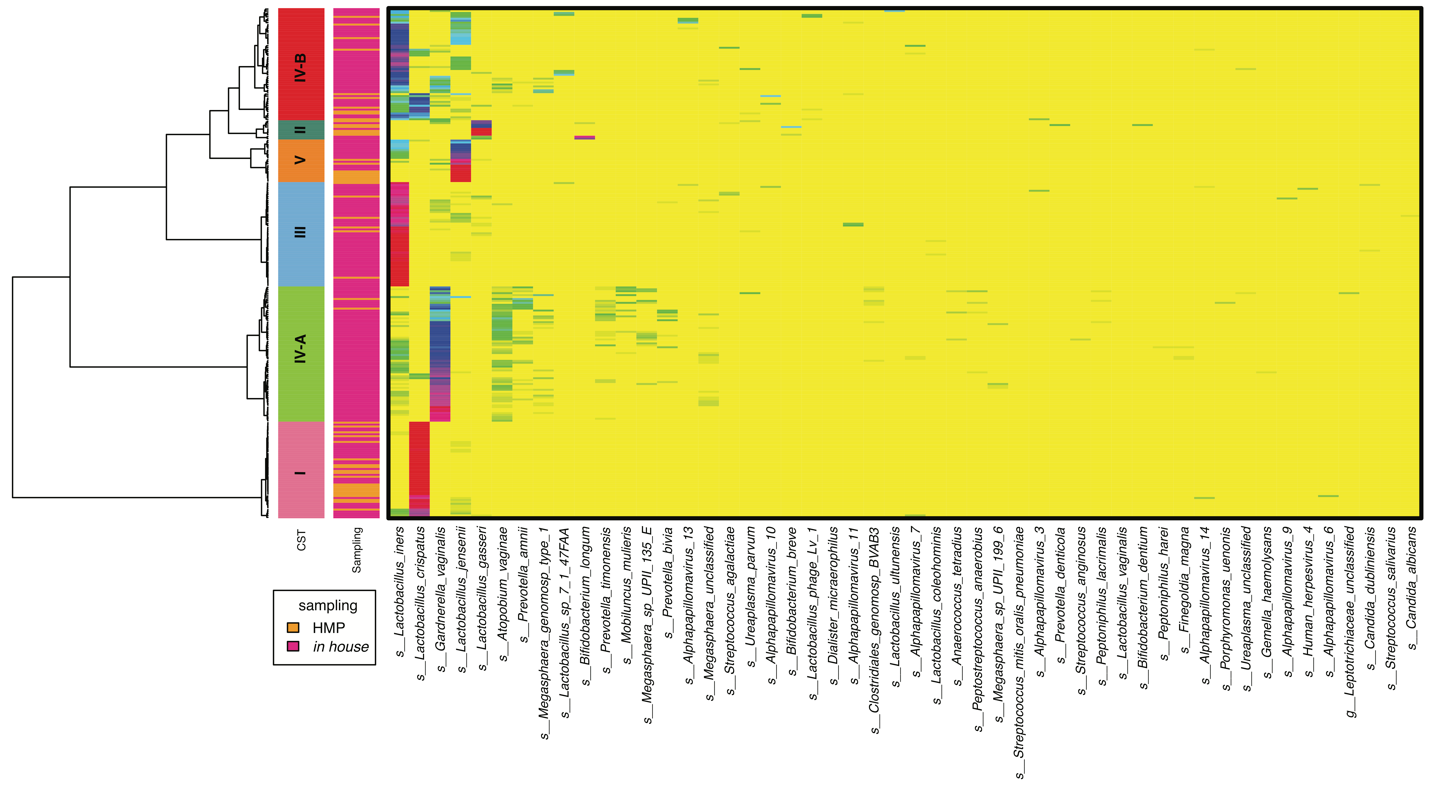
**Supplementary Fig. 2 |** Heatmap of relative abundance of the 50 most abundant phylotypes in the vaginal metagenomes used in this study. Ward linkage clustering is used to clusters samples based on their Jensen-Shannon distance calculated in the *vegan* package in R [100] according to the previous naming convention [29]. The sidebars indicate CSTs and gene richness category, respectively. Gene richness categories include high gene count (HGC) and low gene count (LGC), defined using the threshold of 10,000 genes per sample.


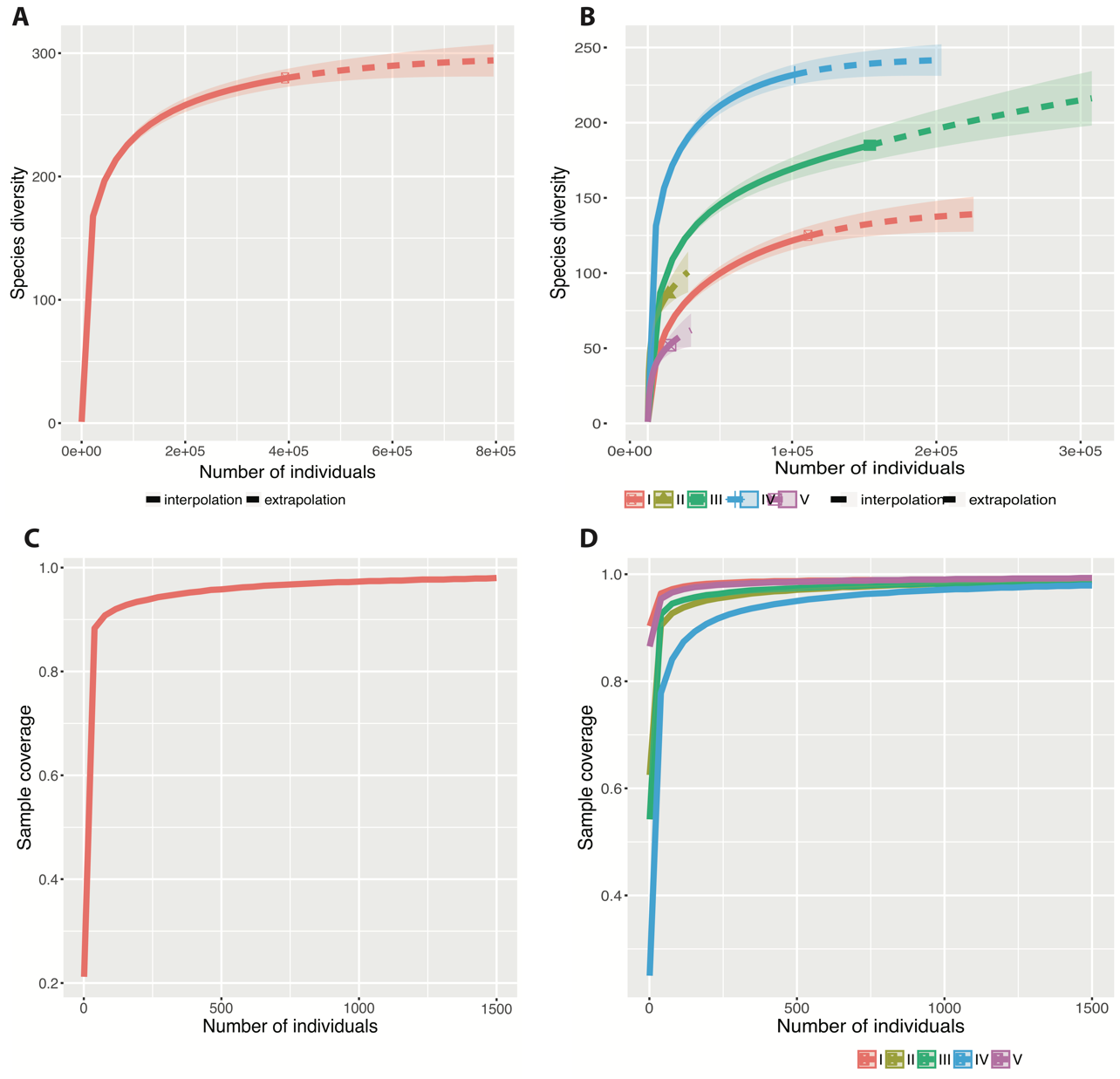
**Supplementary Fig. 3** | Vaginal community accumulation curves and diversity estimate. (A) Accumulative diversity estimates with respect to sample size, for rarefied and extrapolated estimates using all samples; (B) accumulative diversity estimates with respect to sample size, for rarefied and extrapolated estimates using samples of different CSTs; (C) diversity estimate with respect to sample coverage, for rarefied and extrapolated estimates using all samples; (D) diversity estimate with respect to sample coverage, for rarefied and extrapolated estimate using samples of different CSTs. Community diversity estimates were computed using R package *iNEXT* [71] and *vegan* [72]. Sampling curve was either rarefied to smaller sample sizes or extrapolated to a larger sample size for species diversity estimate.


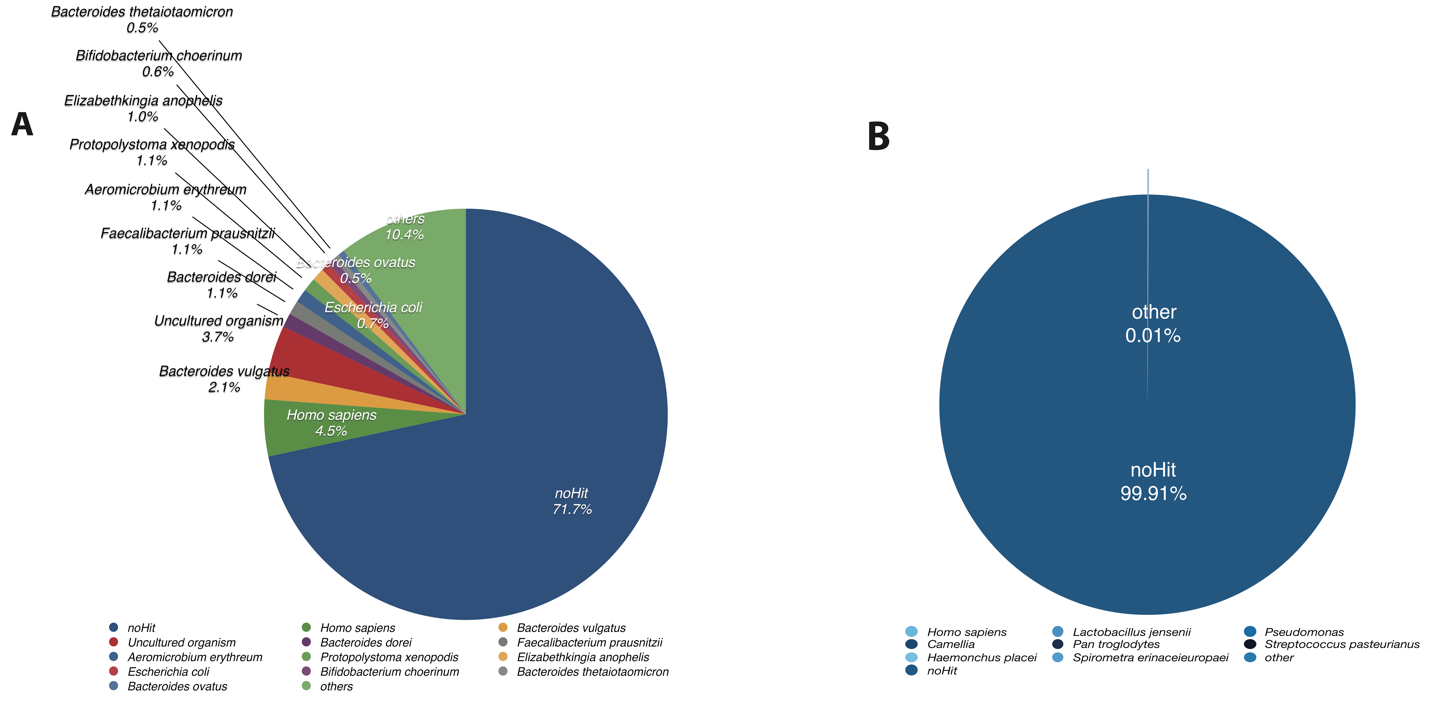
**Supplementary Fig. 4** | Pie chart taxonomic distribution of reads that failed to map on VIRGO for vaginal metagenomes of African women from Gosmann *et al.* [30] in **A** and of Chinese women from [31] in **B**. The unmapped reads were compared to GenBank nt database [97] using BLASTN.


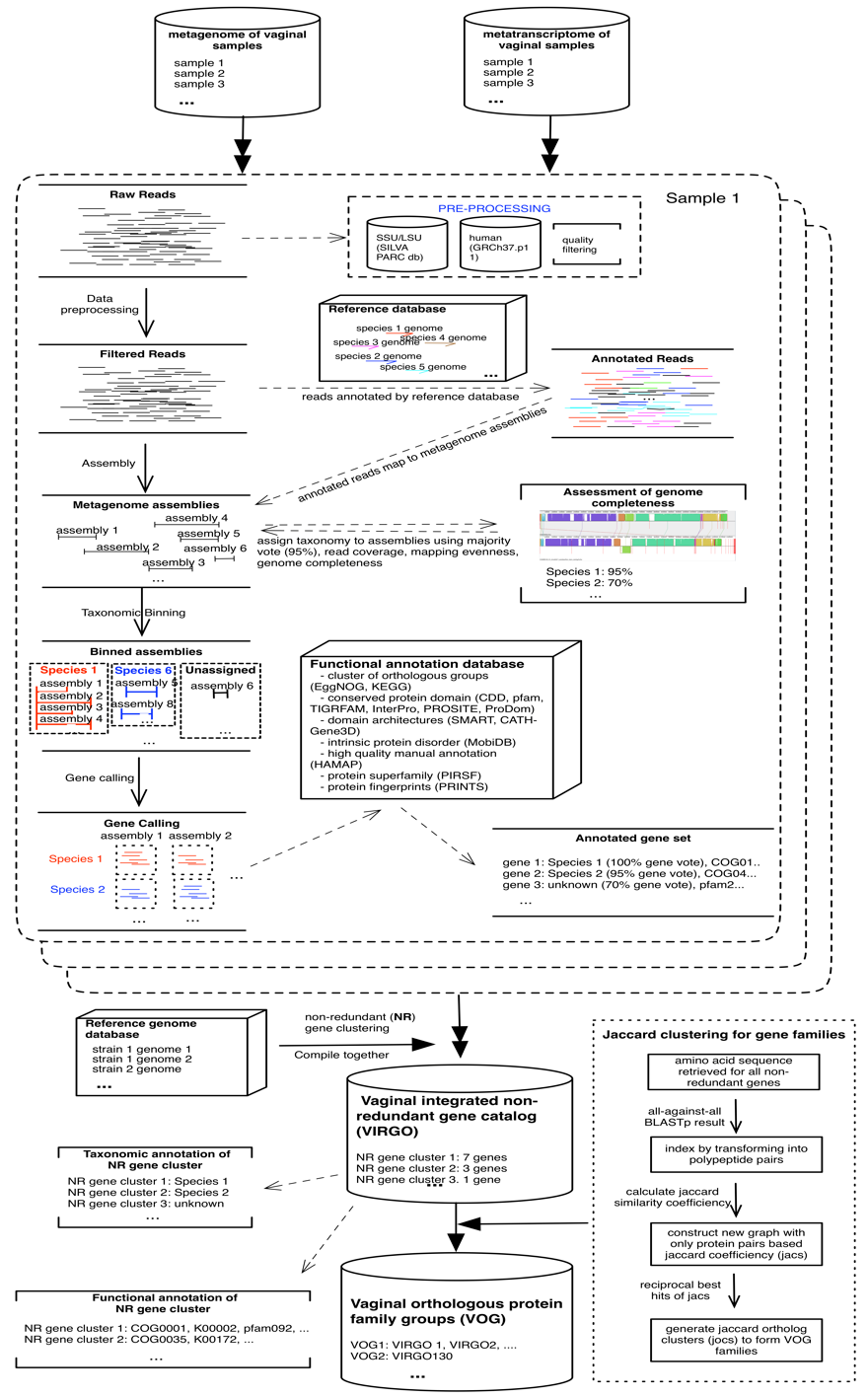


**Supplementary Fig. 5** | Pipeline for data processing and integration for the construction of the human vaginal integrated non-redundant gene catalogue (VIRGO) and vaginal orthologous protein family groups (VOG). Metagenomes from 264 vaginal metagenomes and 416 genomes of urogenital isolates were processed, that including 212 *in-house* sequenced vaginal metagenomes. The procedures include pre-processing to remove human contaminates, quality assessment, metagenome assembly, gene calling, functional and taxonomic annotation, gene clustering based on nucleotide sequencing similarity to form VIRGO, and Jaccard index coefficiency clustering of amino acid sequences to form VOG.


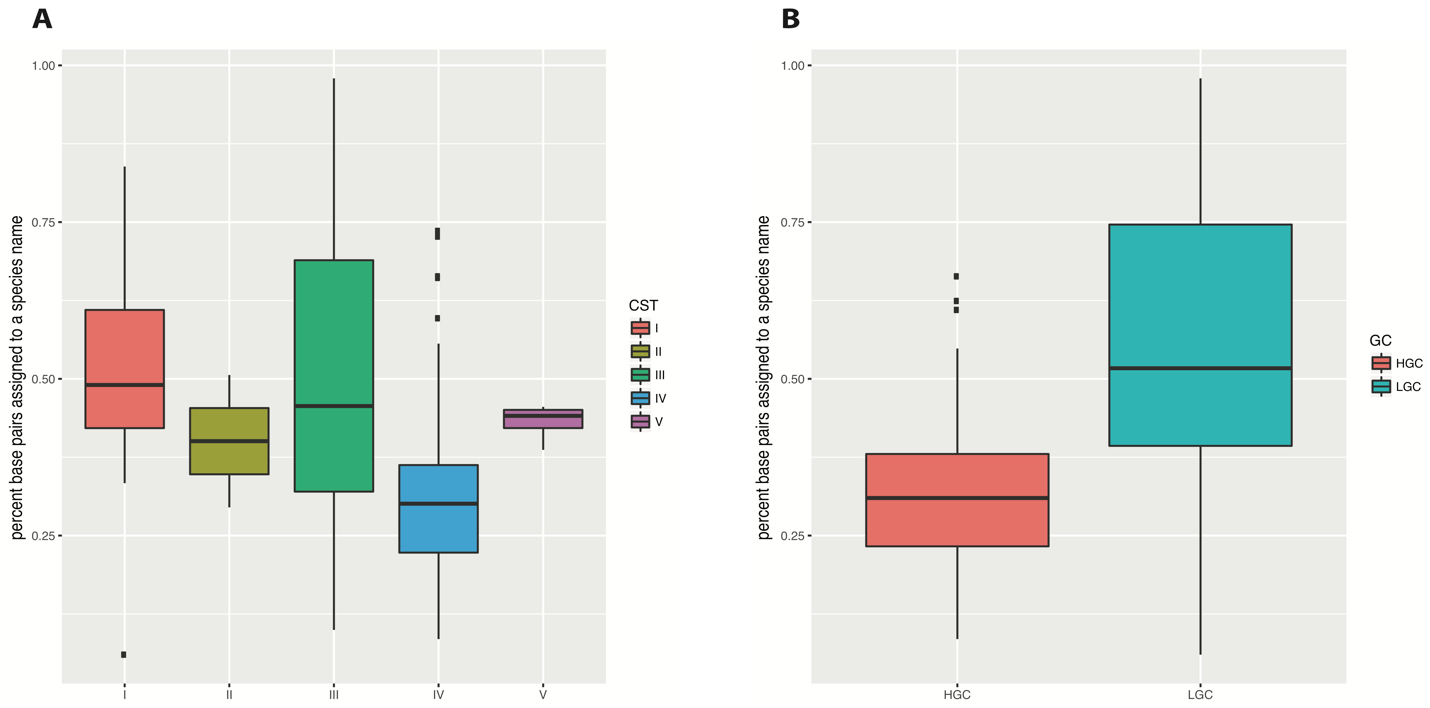
**Supplementary Fig. 6** | Proportion of the assembly length assigned taxonomically from the samples (**A**) among different community state types (CSTs) and (**B**) between different gene richness category. CSTs were defined as previously according to the composition and structure of the microbial community [29]. Gene richness category includes high gene count (HGC) and low gene count (LGC), defined using the threshold of 10,000 genes per sample.


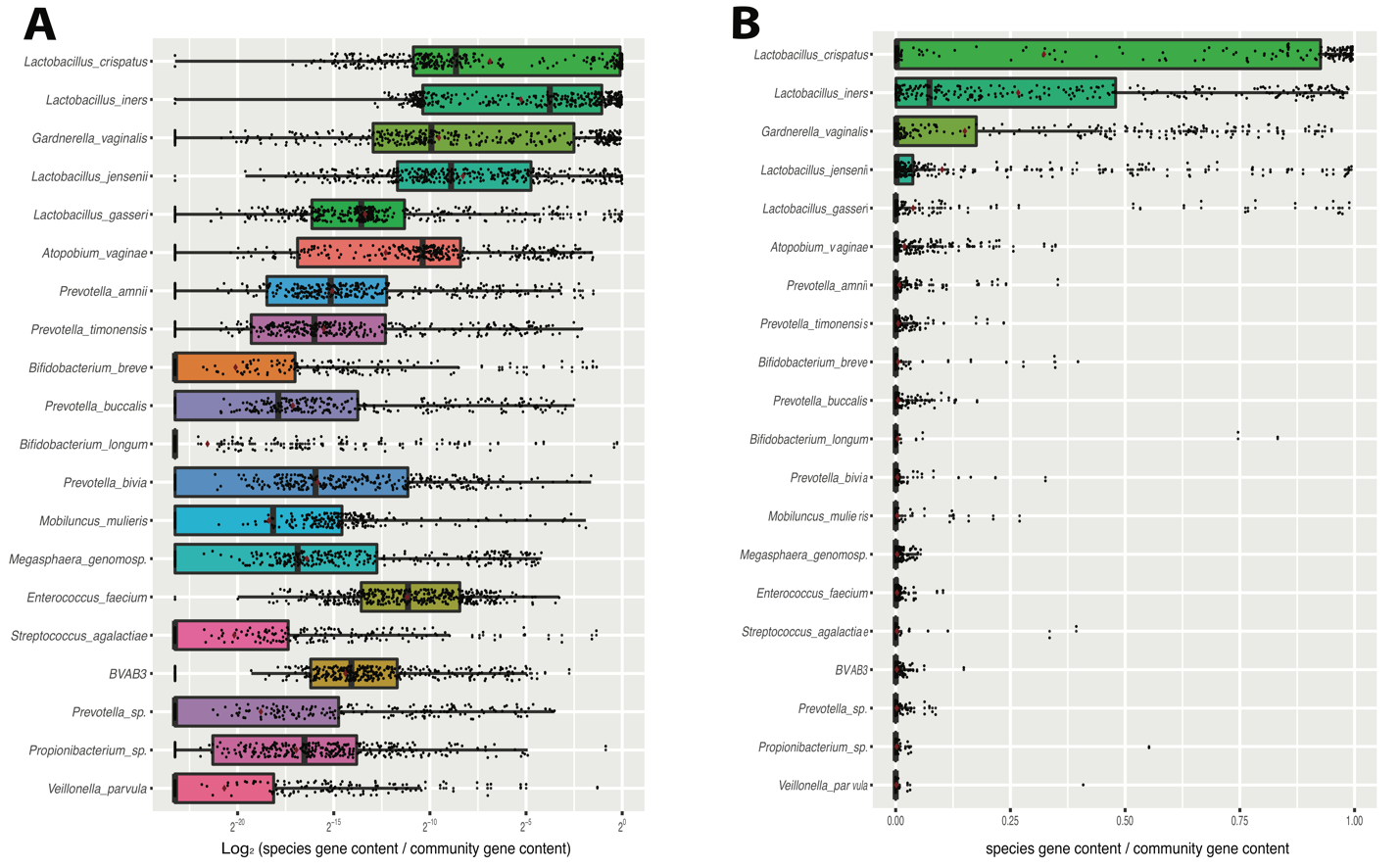
**Supplementary Fig. 7** | Top 20 species with the most abundant gene content in VIRGO. The ratio of the gene content of a species over the entire community in log base 2 **(A)**, and untransformed **(B)**. Plotted are interquartile ranges (IQRs, boxes), medians (line in box), and mean (red diamond).


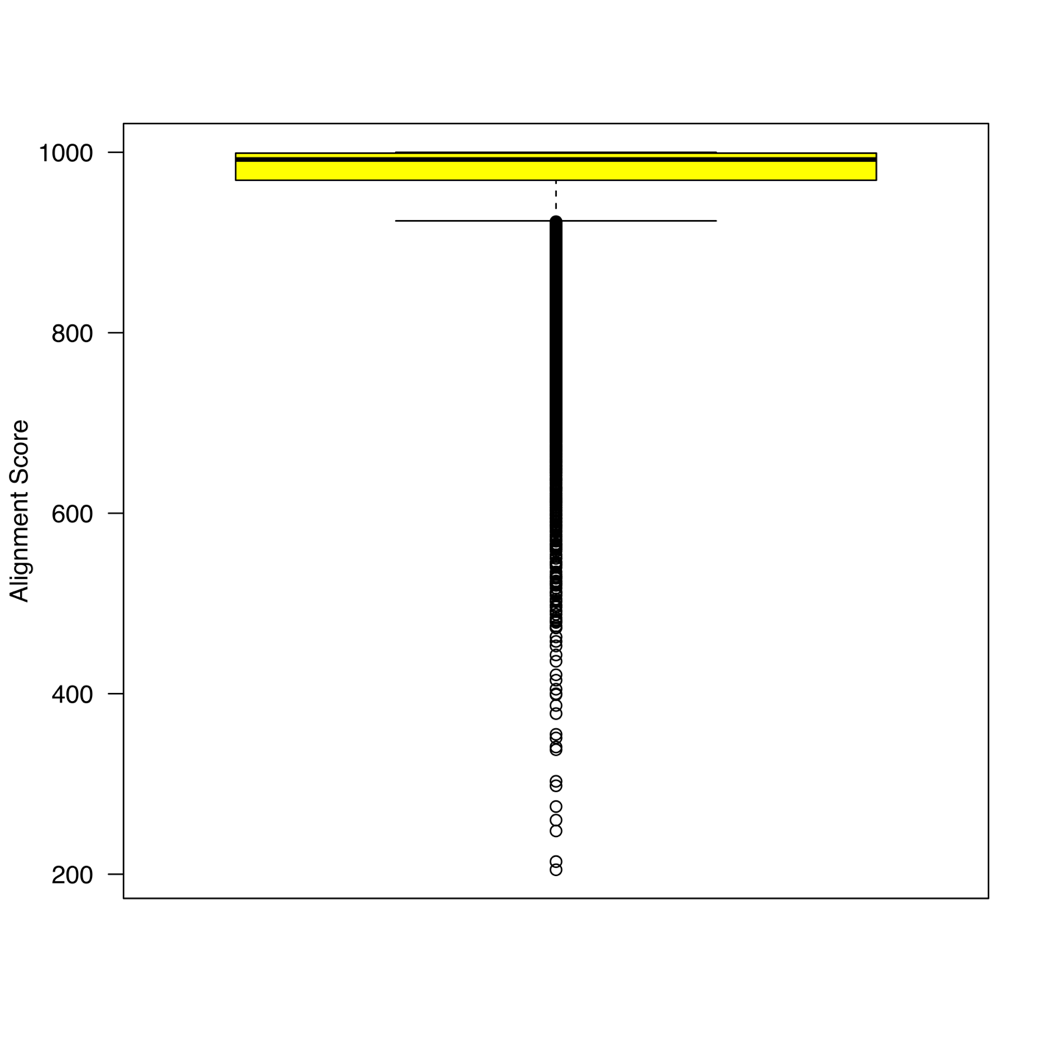


**Supplementary Fig. 8** | Boxplot of the alignment scores of Jaccard orthologous clusters (JOCs) with multiple members. The alignment program T-Coffee [95] was used to access the alignment quality using alignment score.


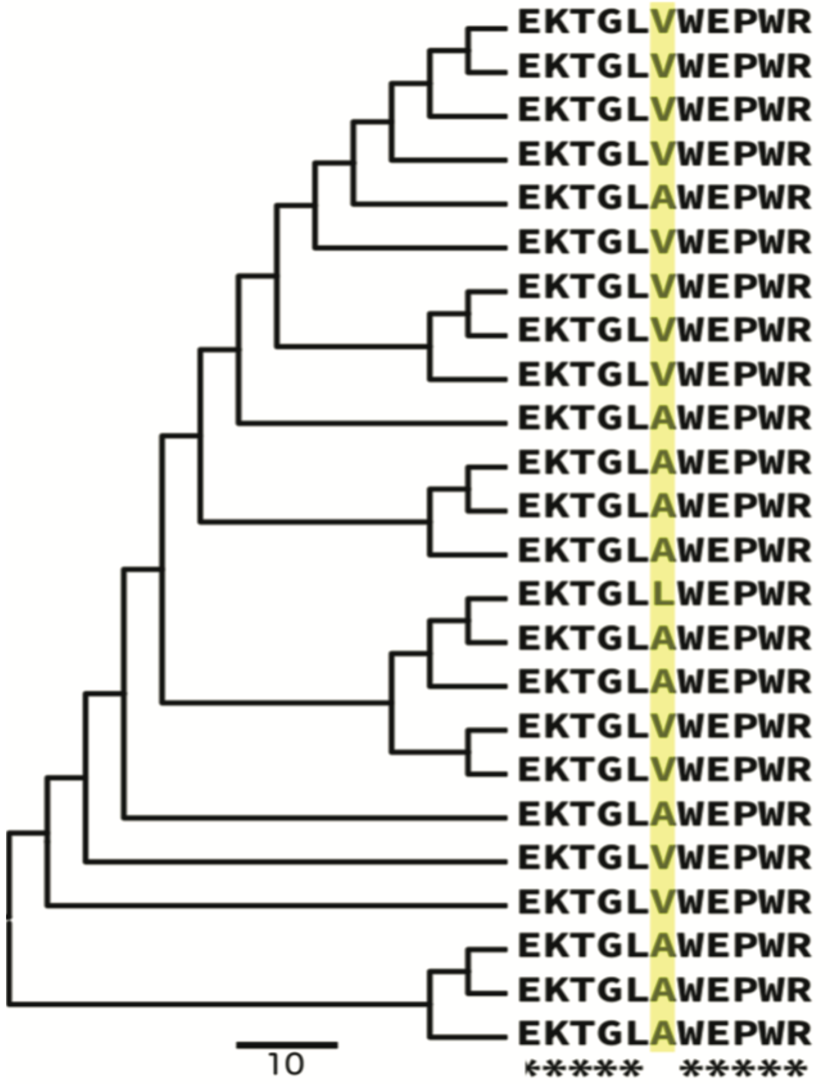


**Supplementary Fig. 9** | Phylogeny that is demonstrative use of VOG to characterize the *G. vaginalis* cholesterol-dependent cytolysin (CDC) protein family. It shows the phylogeny of CDC-containing protein and alignment of domain 4 of the CDCs that is generally well conserved but contains a single divergent site, highlighted in yellow [38].


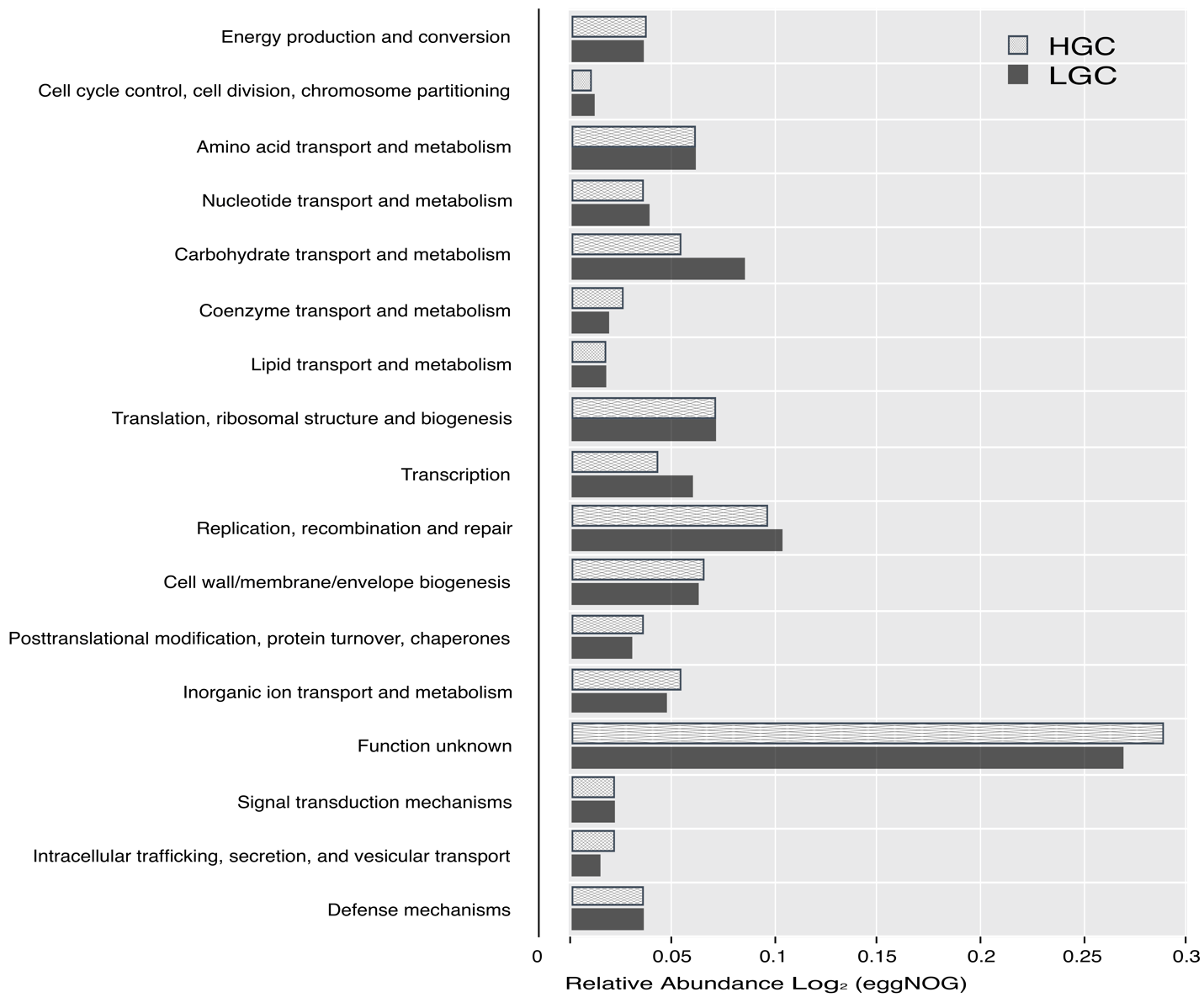
**Supplementary Fig. 10** | Association plot of functional distribution of different gene count categories in vaginal microbiome. Functional category was defined using EggNOG (v4.5) [77] functional category. A Cohen-Friendly association plot [101, 102] was produced in statistical package *vcd* in R [103] to indicate deviations to indicate deviations from independence of CSTs and functional distribution. Mosaics display was shown, where the cells are shaded in proportion to standardized residuals, where the positive value (blue) is the observed frequency is substantially greater than would be found under independence, and the negative value (red) indicates cells which occur less often than under independence.


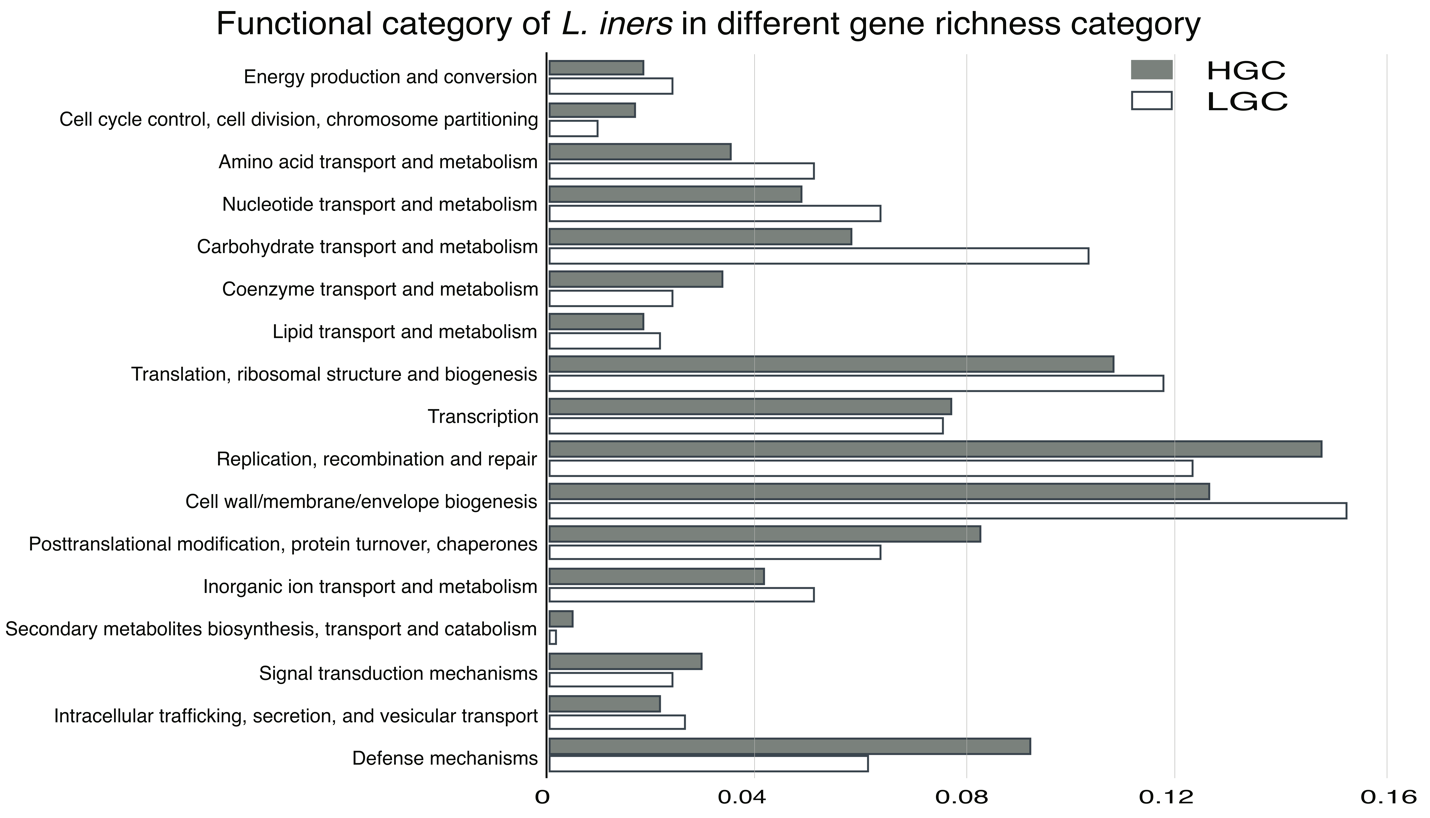
**Supplementary Fig. 11** | Functional category of *L. iners* in different gene richness categories. Functional category was defined using EggNOG (v4.5) [77] functional category.


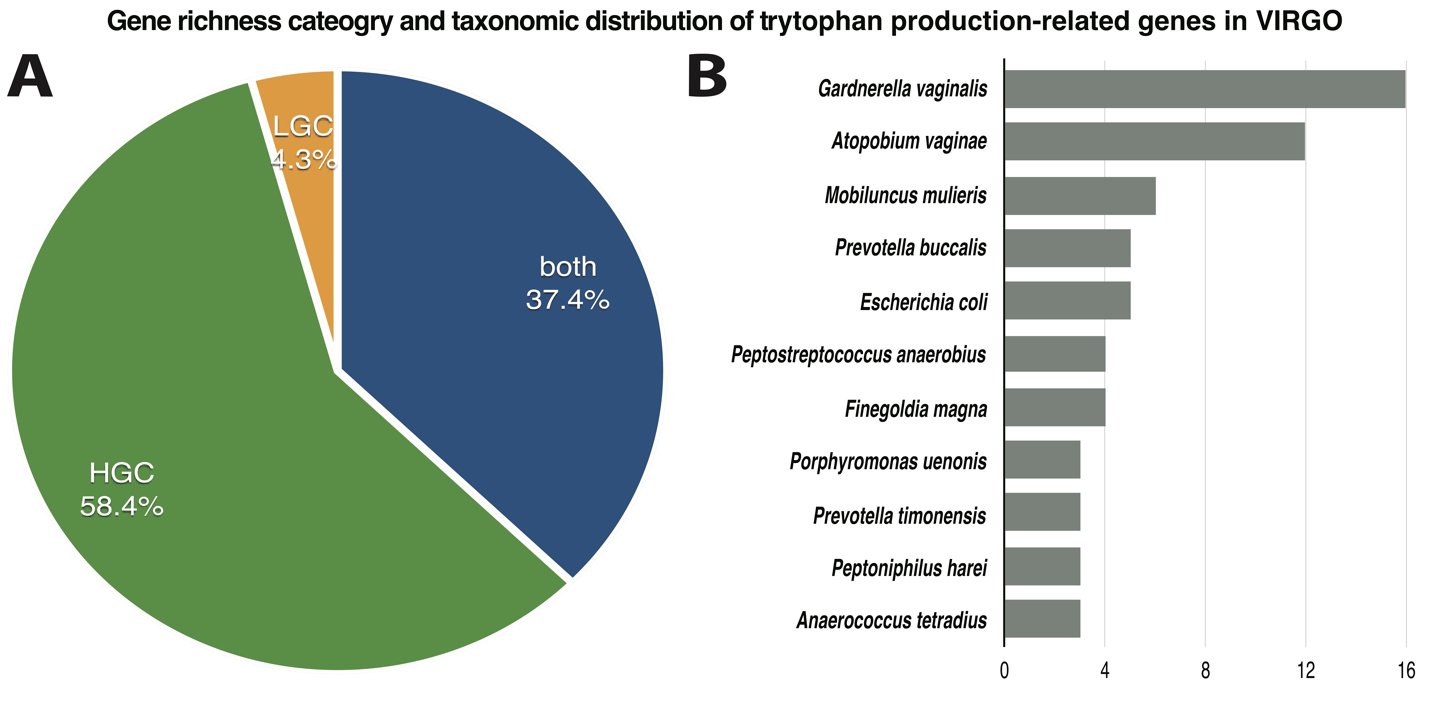


**Supplementary Fig. 12** | Gene richness category and taxonomic distribution of tryptophan production-related genes in VIRGO. (**A**) Pie chart of the percentage of tryptophan production-related genes in different gene richness categories of HGC or LGC. (**B**) The top 10 most affiliated taxonomic groups of the tryptophan production-related genes.

**(A)** *L. iners*


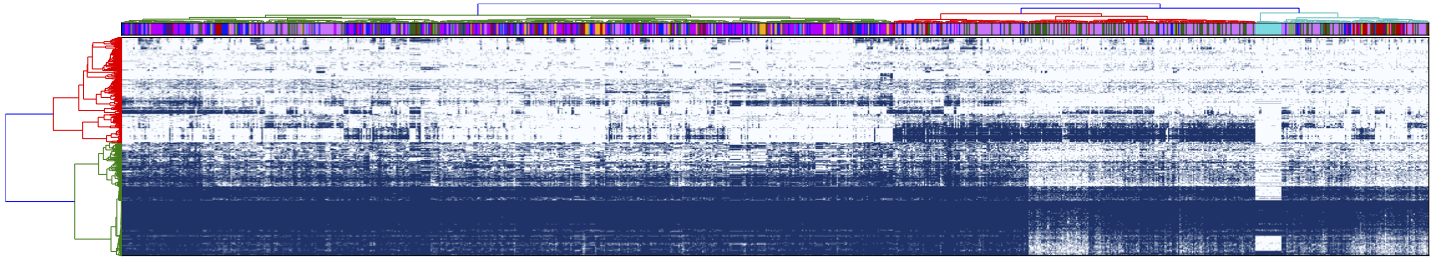

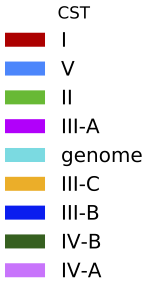


**Supplementary Fig. 13** | Heatmap includes gene prevalence profiling of available genomes of vaginal isolates and VIRGO-characterized metagenomes for (**A**) *L. iners,* (**B**) *L. jensenii,* (**C**) *G. vaginalis*, (**D**) *A. vaginae* and (**E**) *P. timonensis*. Hierarchical clustering of the profiles was performed using ward linkage based on Jaccard similarity coefficient. CSTs were defined as previously according to the composition and structure of the microbial community [29].

**(B)** *L. jensenii*


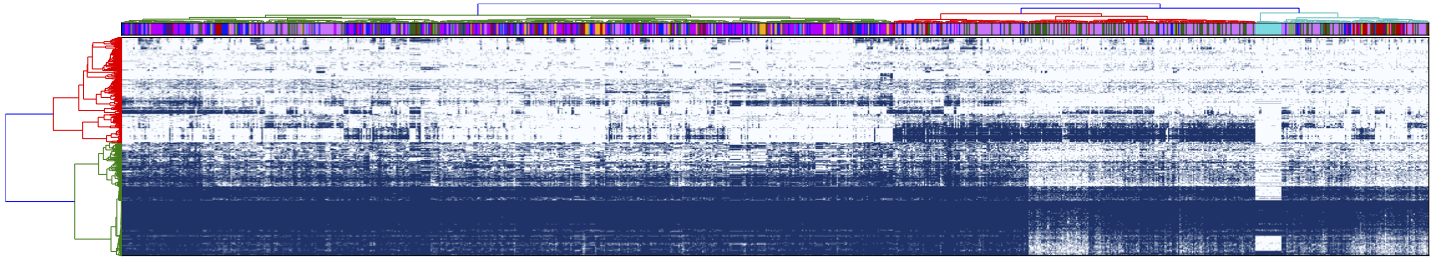


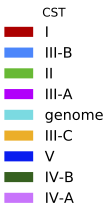


(**C**) *G. vaginalis*


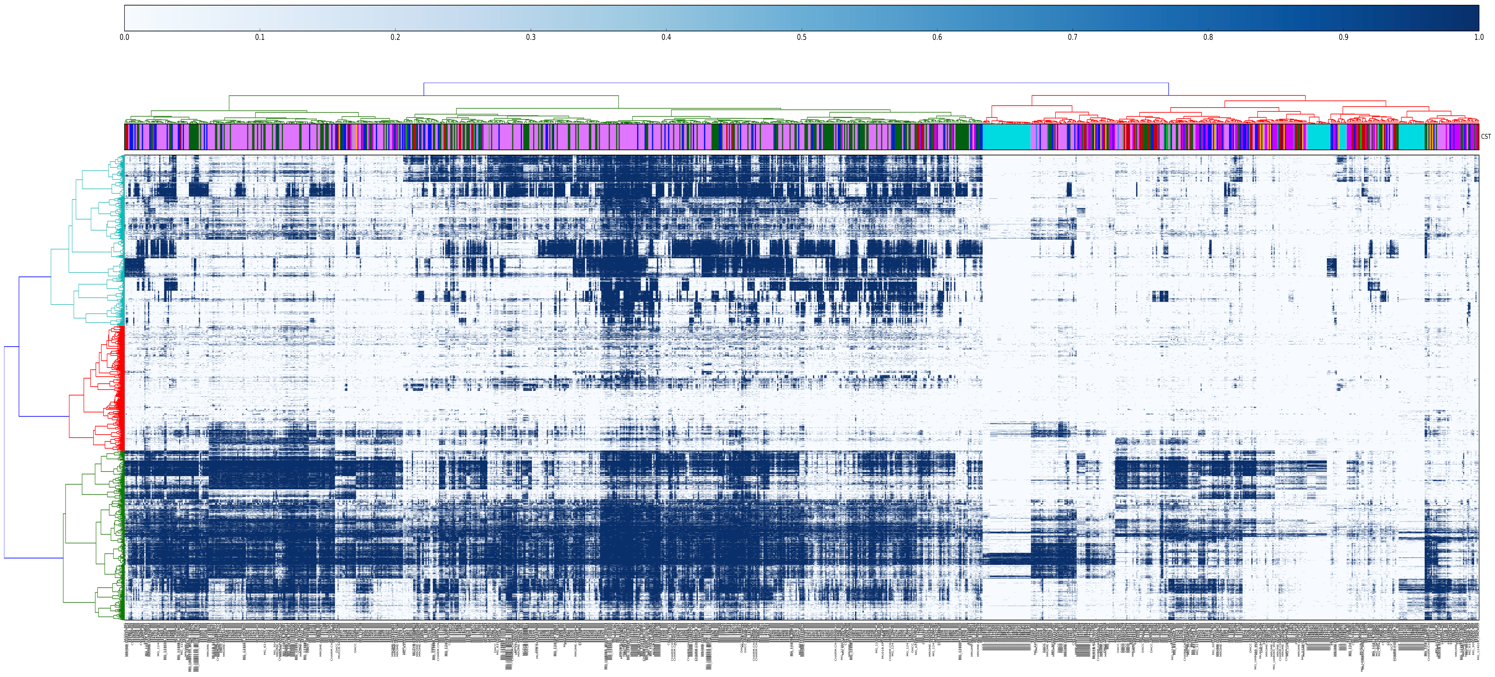


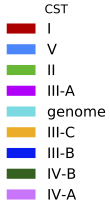


(**D**) *A. vaginae*


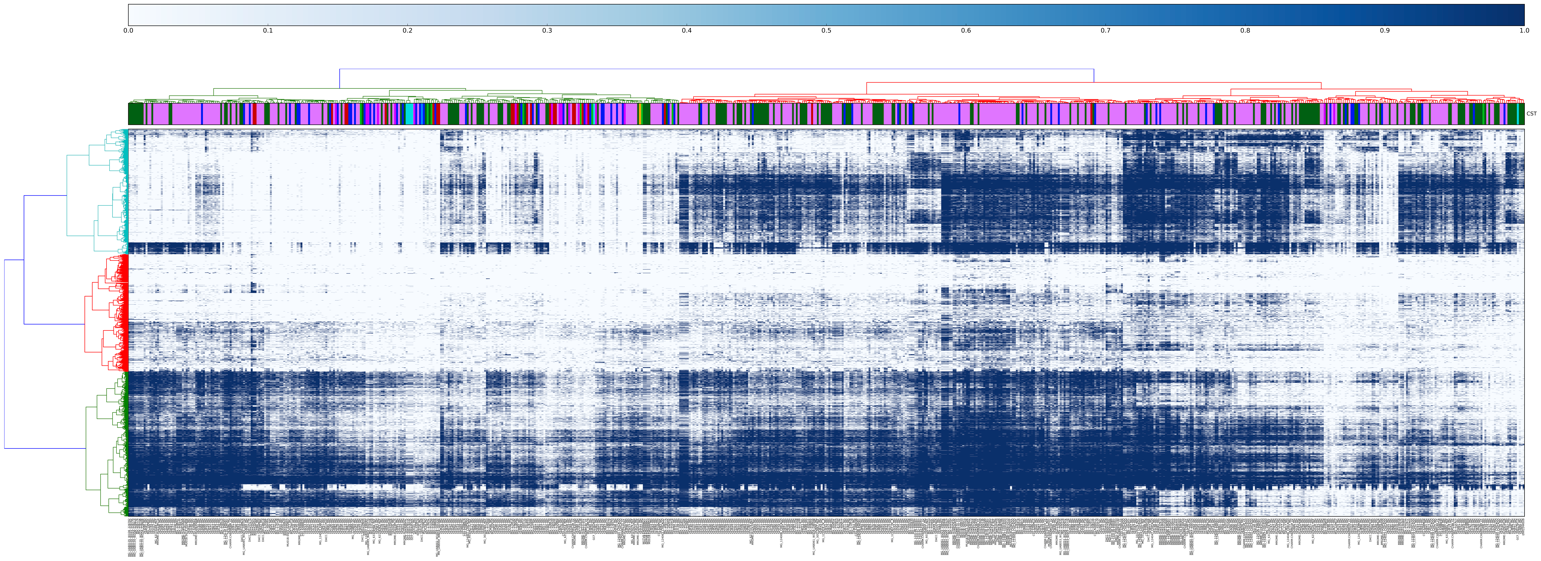


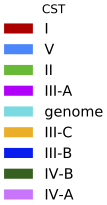


(**E**) *P. timonensis*


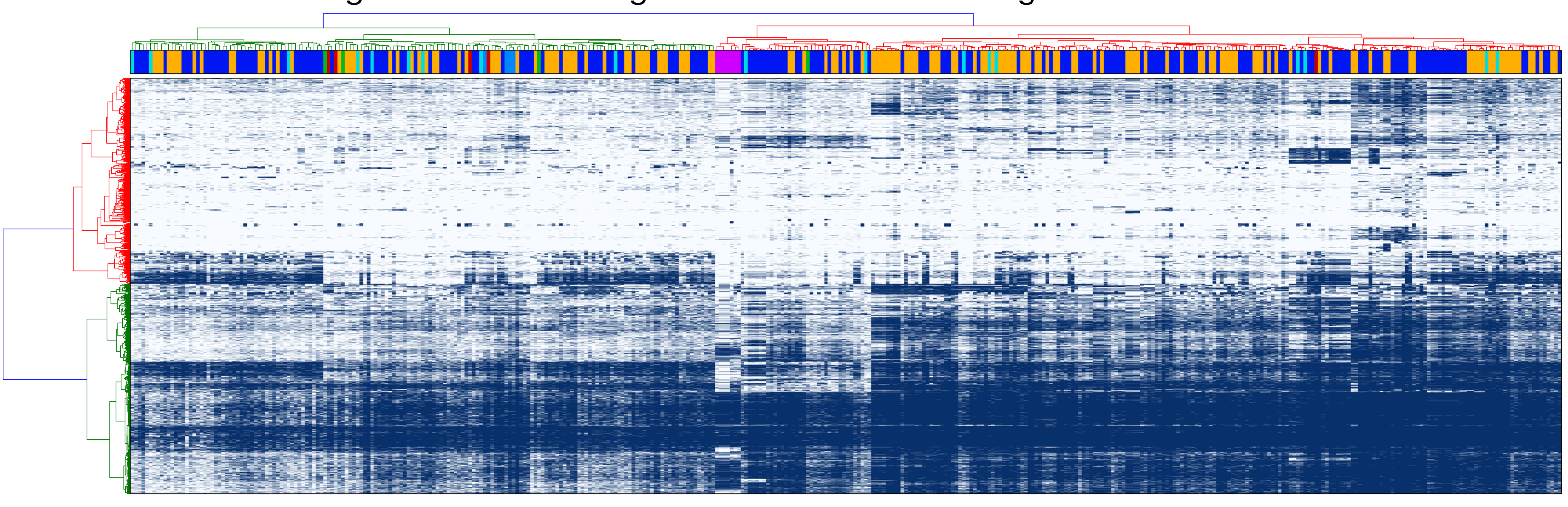


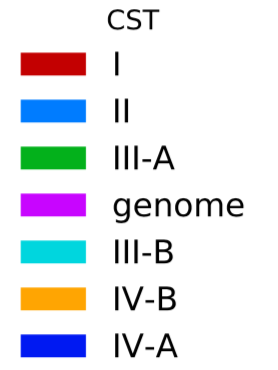

Supplement: Supplementary file 1 — Supplementary Information [file 41467_2020_14677_MOESM1_ESM.docx]
