# Supplementary material for: A comprehensive non-redundant gene catalog reveals extensive within-community intraspecies diversity in the human vagina
Source: Nat Commun. 2020 Feb 26;11:940. doi: 10.1038/s41467-020-14677-3 (PMC7044274; doi:10.1038/s41467-020-14677-3)
Supplement: Supplementary file 3 — Description of Additional Supplementary Files [file 41467_2020_14677_MOESM3_ESM.docx]

**Description of Additional Supplementary Files**

## Filename: Supplementary Data 1

**Description:** Statistics of the sequence reads, including 211 in-house sequenced metagenomes, 53 metagenomes from HMP DACC database, 277 genomes of bacteria isolated from the vagina, reproductive or urinary systems deposited in GenBank and 139 urogenital bacterial genomes from the HMP DACC database used to compile the VIRGO database. The assembly statistics includes assembled base pairs, total number of contigs, N50, mean and median length, and other statistics.

**Filename: Supplementary Data 2**.

**Description:** OTUs table for all metagenomes included in VIRGO. Taxonomic profiling was conducted in MetaPhlAn version 2^19^. Community state types were defined as previously according to the composition and structure of the microbial community^21^. A total of 312 bacterial species that were present in at ≥ 0.01% relative abundance are shown.

**Filename Supplementary Data 3**.

**Description:** Statistics of the complete and subsets of the sequence contigs used to build VIRGO, including reference datasets: i) complete VIRGO database, ii) 212 in-house sequenced vaginal metagenomes, iii) 53 HMP DACC vaginal metagenomes ^24^, iv) all HMP urogenital reference genomes, v) 277 genomes of bacteria isolated from vagina, reproductive or urinary system deposited in GenBank, and vi) 139 genomes of urogenital bacteria from HMP DACC database ^11^.

**Supplementary Data 4**.

**Description:** Counts of the non-redundant genes in VIRGO by taxonomic groups in both species and genera.

**Supplementary Data 5**.

**Description:** 40 genome sequences of fungal vaginal species used in this study, and the abundance of detected fungal and phage genes in the 1,507 metagenomes analyzed in this study.

**Filename: Supplementary Data 6.**

**Description:** Annotation of each protein in a Jaccard orthologous clusters (JOCs) associated with vaginolysin biosynthesis. This JOCs was in one protein family in VOG that contains multiple genes.

**Filename: Supplementary Data 7.**

**Description:** Examples of cell surface-associated proteins of *L. iners*. Two Jaccard orthologous clusters (JOCs) involved in this function were retrieved from VIRGO. (A) the JOC that has the LPXTG motif; (B) the JOC that has the motif YSIRK.

**Filename: Supplementary Data 8.**

**Description:** Number of non-redundant genes identified in a metagenome and the associated sequencing depth for samples in different CSTs.

**Filename: Supplementary Data 9.**

**Description:** Examples of tryptophan production-related gene identified using VIRGO. It includes three essentials genes:Tryptophanase (tnaA), Tryptophan synthase beta chain (trpB), and Tryptophanyl-tRNA synthetase (trpS) to demonstrate the profiling of a specific function of interest and its taxonomic distribution.

**Filename: Supplementary Data 10.**

**Description:** Summary of the 7 vaginal bacterial species for which the gene content was characterized using VIRGO to determine the diversity of individual populations. It includes four Lactobacillus species (*L. crispatus*, *L. iners*, *L. jensenii*, and *L. gasseri*), as well as three additional species common to the vagina (*G. vaginalis*, *A. vaginae* and *P. timonensis*). Reads mapping was performed using 1,507 in-house and publicly available vaginal metagenomes to VIRGO. Metagenomes that contained at least 80% of their average genome’s number of coding genes were included. Abbr: Av: *A. vaginae*; Gv: *G. vaginalis*; Pt: *P. timonensis*; Lc: *L. crispatus*; Li: *L. iners*; Lj: *L. jensenii*; Lg: *L. gasseri*.

**Filename: Supplementary Data 11.**

**Description:** List of accession numbers for genomes of the four *Lactobacillus* species including *L. crispatus*, *L. iners*, *L. jensenii*, and *L. gasseri* and three species including *G. vaginalis*, *A. vaginae* and *P. timonensis* used in intraspecies analyses.

**Filename: Supplementary Data 12.**

**Description:** Taxonomic distribution of pullulanase domain-containing proteins included in VIRGO.
